# Effects of *Melaleuca alternifolia* Chell (Tea Tree) and *Eucalyptus globulus* Labill. Essential Oils on Antibiotic-Resistant Bacterial Biofilms

**DOI:** 10.3390/molecules28041671

**Published:** 2023-02-09

**Authors:** Ramona Iseppi, Martina Mariani, Stefania Benvenuti, Eleonora Truzzi, Patrizia Messi

**Affiliations:** 1 Department of Life Sciences, University of Modena and Reggio Emilia, Via G. Campi 287, 41125 Modena, Italy; 2 Burn Intensive Care Unit, Hospital A. Cardarelli, Via A. Cardarelli 9, 80131 Naples, Italy

**Keywords:** antibiotic resistant bacteria (ARB), essential oils (EOs), antibiotics, synergy association, anti-biofilm activity, MRSA, methicillin-resistant *Staphylococcus aureus*, VRE, vancomycin-resistant enterococci, ESBL, extended spectrum β-lactamase *Escherichia coli*

## Abstract

In the present investigation, the anti-biofilm potential of two essential oils (EOs), *Melaleuca alternifolia* Chell (Tea-Tree) (TTO) and *Eucalyptus globulus* Labill. (EEO) was characterized and tested “in vitro” against both mature biofilms and biofilms in the process of formation, produced by strains belonging to three main categories of antibiotic resistant bacteria (ARB): Vancomycin-resistant enterococci (VRE), methicillin-resistant *Staphylococcus aureus* (MRSA) and broad-spectrum β-lactamase-producing *Escherichia coli* (ESBL). The study was carried out in 96-well microtiter-plates using EOs alone, in association with each other and in combination with antibiotics against both single and multi-species biofilm. The study demonstrated the ability of TTO and EEO to counteract the ARB strains in sessile form, with promising results in particular against the biofilm in formation. Mature biofilm by ESBL *E. coli* was the most sensitive in the results from the quantification study of viable cells performed in multi-species biofilms. Lastly, in all tests, carried out using TTO/EEO associations and EOs/antibiotic combinations, the synergistic effect which emerged from the FIC-index has been confirmed, and both the reduction of biofilm in formation, and the removal of mature structure was obtained at very low concentrations, with values from 4 to >512-fold lower than the minimum inhibitory concentration (MIC) of the single compounds.

## 1. Introduction

In recent years, bacteria resistant to multiple pharmacological agents have steadily increased and the infections caused by antibiotic-resistant bacteria (ARB) are an important global problem [1], with a significant impact both clinically and economically. Antimicrobial resistance is currently considered a major concern worldwide, and the World Health Organization recently included it among the top 10 threats to human health [2]. One of the main causes of antibiotic resistance is due to the abuse of antibiotics for therapeutic purposes in humans, but also to their massive use in the veterinary field, leading to the development of resistance to most classes of antibiotics. The environment where it is easier to detect ARB strains is the hospital, where the selective pressure that favors them is greater, making them responsible for infections that are difficult to treat with common antibiotics [3]. Hospital Acquired Infections (HAI) are one of the most frequent and serious complications of health care. Together with the indiscriminate and often incorrect use of antibiotics, environmental contamination plays an important role, especially when the bacteria responsible for HAI are found within biofilms. The biofilm is defined as a structured community of microorganisms, enclosed in a self-produced polymeric matrix, adhering to an inert or living surface in an aqueous medium. Biofilms are very heterogeneous entities and can consist of a single microbial species or a set of microorganisms (including fungi, algae, and protozoa). The formation of biofilm requires interactions, communication and coordination between the different microbial species that constitute it [4], and this feature allows microbial cells to organize themselves in communities to continuously adapt to extreme conditions. Biofilms can be formed on biotic and abiotic surfaces, such as hydrated inorganic surfaces, solid surfaces of medical devices (catheters, prostheses, implants), epithelia, mucous membranes, and dental plaques. Structurally, the basic unit of the biofilm is the microcolony [5]. One of the biological characteristics that distinguish biofilm from planktonic populations is the presence of a self-produced extracellular matrix (EPS, extracellular polymeric substance), composed of a mix of polysaccharides, water, ions, DNA, and proteins released by the biofilm’s bacteria that form a “supramolecular” structure [6]. The biofilm matrix, therefore, represents a barrier for antimicrobial agents, both blocking their spread and interacting chemically with the drug. Bacteria also exhibit remarkable plasticity and can perceive and respond to many external stimuli, adapting to most environments [7]. Biofilms show resistance to antimicrobial agents 10 to 1000 times higher than that shown by the same cells in planktonic form [8]. The antimicrobial resistance of the biofilm is phenotypic, i.e., it is not caused by mutations or carried by plasmids and transposons (even if the nearness between different microbial species favors genetic exchange through the conjugation mechanism), but is mainly due to its structural characteristics, and to the metabolic and functional properties of the bacterial cells that are part of it. There are several mechanisms proposed to explain the antimicrobial resistance of the biofilm. It could, in fact, be related (i) to the extracellular matrix, which constitutes a mechanical barrier to drug penetration, (ii) to the reduction of their growth because they are able to easily obtain oxygen, metabolites and nutrients through the favorable structure that hosts them [9], with consequent slowdown of cellular processes such as the synthesis of DNA, proteins and peptidoglycan, molecular targets of many classes of antibiotics [10]; (iii) to the presence in the biofilm of a subpopulation of viable but not cultivable state (VBNC), a condition of quiescence that greatly increases their resistance [11]. These important issues in antibiotics resistance, linked to the hospital environment, where biofilm-related diseases are frequent [12,13], impose the need to expand the search for new antimicrobial substances. However, in the last 40 years few new classes of antibiotics have been discovered. One of the reasons for this decline is the difficulty in finding new chemical entities that are simultaneously active and not toxic [14]. A growing interest by scientific communities is directed towards a number of natural products which, in recent studies, have shown themselves able to combat this phenomenon. Essential oils (OEs) extracted from plants are considered a possible source of new antibacterial molecules with a broad spectrum of action different from traditional antibiotics and could represent a valid solution to the problem. OEs or aromatic plant essences are volatile and contain fragrant substances with an oil of a consistency typical of the plant from which it is produced. They are synthesized by all the organs of the plant (buds, flowers, leaves, stems, twigs, seeds, fruits, roots, wood, or bark) and are stored in secretory cells, cavities, channels, epidermal cells or glandular trichomes. The interest in EOs has increased over time, mainly because of their biological properties. Essential oils are already used in the perfume industry, as taste and odor modifiers, in aromatherapy, as insect and animal repellents, and in pharmaceutical preparations. However, the most important aspect concerns their antimicrobial activity, on which numerous studies have focused, especially regarding antibacterial [15,16] and antifungal [17] properties. Furthermore, some essential oils have shown insecticidal [18], antiviral [19] and antiparasitic properties [20]. Studies have reported the anti-biofilm activity of plant extracts [21,22], such *Melaleuca alternifolia* (Tea-tree) and *Eucalyptus globulus* essential oils. The inhibition activity of Tea-tree oil on biofilm was observed against *Staphylococcus aureus* [23], *Escherichia coli* [24], and *Candida albicans* [25]. *Eucalyptus globulus* showed 1,8-cineole as its major constituent [26], and exhibited anti-biofilm activity against methicillin-resistant *Staphylococcus aureus* (MRSA) isolates [27].

The aim of the present investigation was to verify the anti-biofilm potential of two essential oils Chell (EOs), *Melaleuca alternifolia* (Tea-tree) (TTO) and *Eucalyptus globulus* Labill. (EEO). The two EOs were tested “in vitro” against both mature biofilms and biofilms in the process of formation, produced by strains belonging to three main categories of antibiotic-resistant bacteria: Vancomycin-resistant enterococci (VRE), methicillin-resistant *Staphylococcus aureus* (MRSA) and broad-spectrum β-lactamase-producing *Escherichia coli* (ESBL). To highlight synergistic interactions between the two types of antimicrobials (natural EOs and synthetic drugs), the activity of the two EOs was determined using them alone and in association with each other, but also in combination with reference antibiotics, to which the three types of bacterial strains were resistant.

## 2. Results

### 2.1. Qualitative and Semi-Quantitative Analysis

*M. alternifolia* and *E. globulus* EOs were phytochemically characterized by means of gas chromatography (GC) and gas chromatography coupled by mass spectrometry (GC-MS). By using retention data, mass spectra, and data reported in the literature, it was possible to identify the analytes in the two samples. More than 95% of the total composition for each EO was characterized (Table 1 and Figures S1 and S2).

As reported in Table 1, and according to NIST 14 (National Institute of Standards and Technology, USA; 14th edition) library database [28], Babushok et al. [29], and Dong et al. [30], TTO showed a composition rich in terpinen-4-ol (43.29%), γ-terpinene (20.16%) and α-terpinene (8.89%). Other peculiar constituents were terpinolene (3.35%), α- terpineol (2.99%), *p*-cymene (2.84%), α-pinene (2.56%) and 1,8-cineole (2.35%) TTO composition was in accordance with the data reported in the literature [31]. Noumi et al. [32] observed that their TTO was rich in terpinen-4-ol (40.44%), γ-terpinene (19.54%), α-terpinene (7.69%), 1,8-cineole (5.20%), *p*-cymene (4.74%), and α-terpineol (3.31%). Brun et al. [33] analyzed ten TTO samples, all with a content from approximately 42% to 48%, γ-terpinene from 18% to 25%, and α-terpinene from 8% to 12%. In our study, the percentages of the major components of TTO are within these ranges, with a lower percentage for α-terpinene than other studies [34,35]. EEO showed a composition in which the major components were 1,8-cineole (58.07%), linalool (12.05%), linalyl acetate (10.95%), camphor (4.39%) and α-pinene (2.33%). The composition of EEO was atypical due to its low percentage of limonene and the high percentages of linalool, linalyl acetate and camphor [36]. The amount of 1,8-cineole showed was similar to that found by other authors, while the content of limonene and α-pinene observed in other studies was higher than those found in this tested EEO [37,38,39].

### 2.2. Determination of the Fractional Inhibitory (FIC) Index

**Figure 1:**
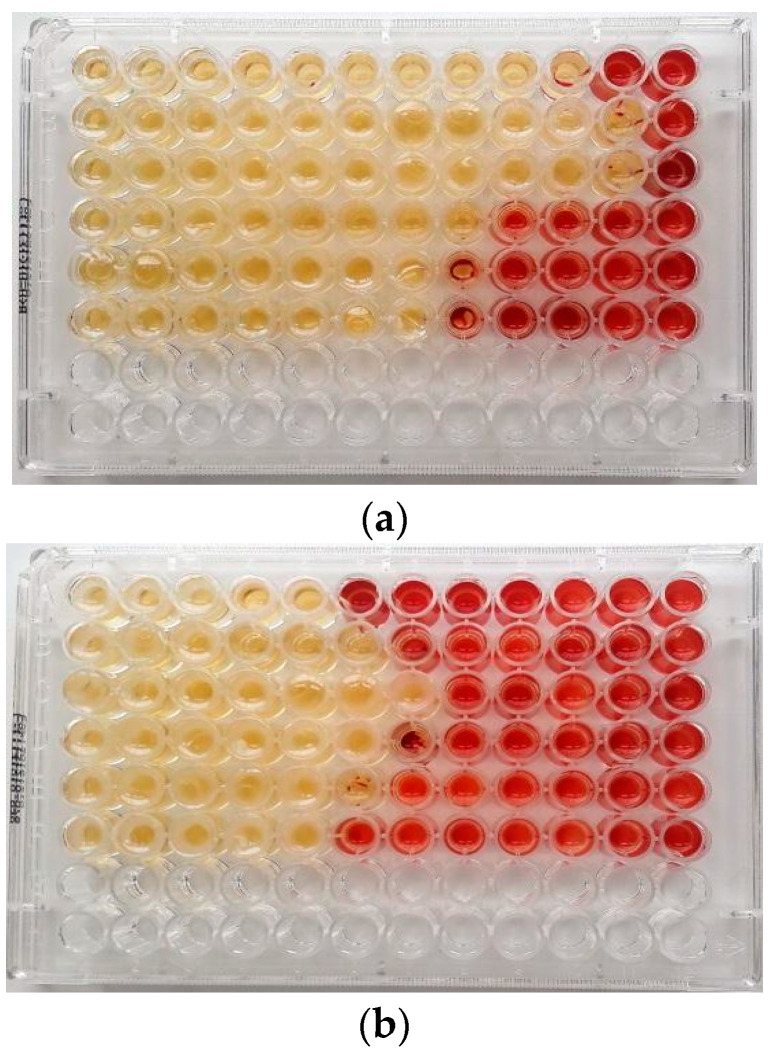
MIC (μg/mL) of (**a**) *Malaleuca alternifolia* Chell. (TTO) and (**b**) *Eucalyptus globulus* Labill. (EEO) EOs determined by the broth microdilution method with addition of 2,3,5-triphenyltetrazolium chloride (TTC) at concentrations of 0.5% against ARB pathogens in planktonic form. Serial dilutions of EOs were added to each well to obtain concentrations ranging from 512 to 0.25 μg/mL (from left to wright). Line 1: *Escherichia coli* 22BT; Line 2: *Escherichia coli* 45DT; Line 3: *Enterococcus faecium* A29; Line 4: *Enterococcus faecalis* VAN3; Line 5: *Staphylococcus aureus* C3; Line 6: *Staphylococcus aureus* O.

Table 2 shows the antibacterial activity of the EOs used both alone (Figure 1a,b), in association and in combination with the antibiotics. The results of the antibacterial activity of EO/EO associations and EO/antibiotic combinations clearly showed a synergistic effect among the active compounds in many determinations, while no antagonistic effects were found, confirming what emerged in our previous investigation [40]. The association between EOs led to an increase in their antibacterial effect, with a contextual decrease in their employed concentrations. As regards the EO/antibiotic combinations, a positive modulation in the reduction of the drug resistance among the pathogenic strains tested was observed. The antibiotic concentrations, when the compound was used together with the EO, were lower than the breakpoint of each ARB specie. In all cases, the anti-biofilm activity was obtained with values from 4 to >500-fold lower than the minimum inhibitory concentration (MIC) of the single compounds. This result is reported both in Table 2 as Drug Lowering Concentration and in the following figures (see below).

### 2.3. Effect of EOs on Mono-Species Biofilm Formation

**Figure 2:**
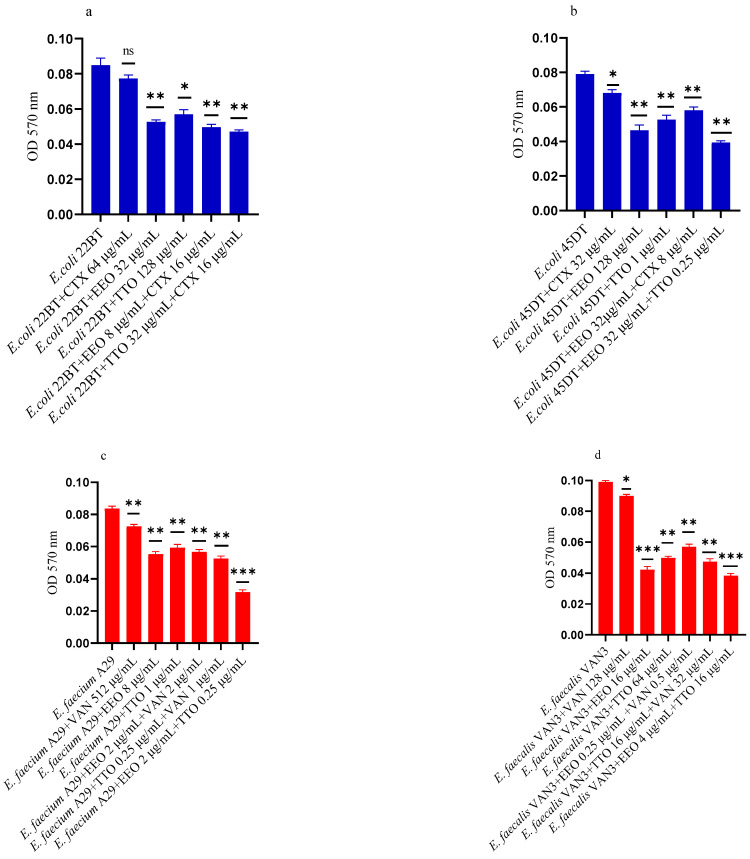

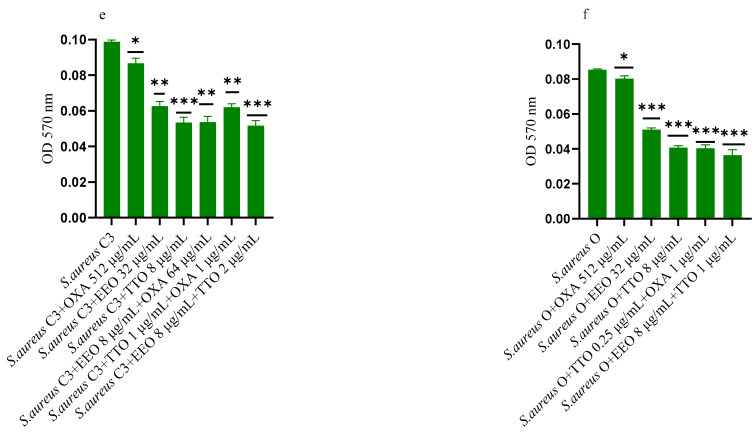
Anti-biofilm formation activity of *Melaleuca alternifolia* (TTO) and *Eucalyptus globulus* (EEO) EOs, of antimicrobials (cefotaxime -CTX-, vancomycin –VAN- and oxacillin-OXA-), and of different combinations/association (antimicrobial/EO and EO/EO) against (**a**) ESBL *Escherichia coli* 22 BT, (**b**) ESBL *Escherichia coli* 45DT, (**c**) VRE *Enterococcus faecium* A29, (**d**) VRE *Enterococcus faecalis* VAN3 (**e**) MRSA *Staphylococcus aureus* C3 and (**f**) MRSA *Staphylococcus aureus* O strains. Results were expressed in optical density (OD) 570 nm as the arithmetic mean of the three determinations. *p*-values of <0.05 (*), *p* < 0.01 (**), *p* < 0.001 (***) were considered significant by *t*-test and ANOVA using statistical program GraphPad Prism 9.2.0 (San Diego, CA, USA). ns stands for not statistically significant.

Figure 2a–f shows the antibacterial activity of reference antibiotics, of single EOs and of most advantageous synergistic mixtures, chosen based on the FIC index, against mono-species formation biofilms. The results demonstrate a promising potential of the compounds to reduce the growth of mono-species biofilms. Against ESBL *E. coli* strains, the activity of all EOs, alone and in combination with cefotaxime, was evident at very low concentrations compared to the MIC of the single compounds. Both EOs showed a good inhibition on *E. coli* biofilm development, in particular EEO (*p* = 0.008 and *p* = 0.0075 for *E. coli* 22BT and *E. coli* 45DT, respectively) (Figure 2a,b), and the TTO/EEO association showed the best result in 52.50% reduction of biofilm formation of *E. coli* 45DT (*p* = 0.0013) (Figure 2b).

A considerable reduction in biofilm growth was also observed against VRE Enterococci (Figure 2c,d), when tested with both the EOs alone and in association/combination. Notably, for both strains, the EEO gave the best result, and the EEO/VAN combination was very effective against the forming biofilm. Once again, for both strains, the treatment with the EEO/TTO association provided the best results with a reduction of 54.88% and 58.76%, respectively (*p* = 0.0009 and *p* = 0.0002 for *E. faecium* A29 and for *E. faecalis* VAN3, respectively).

TTO and its association with EEO showed the best inhibitory activity (reduction of 40.82% and 36.73%, respectively) against the forming biofilm of *S. aureus* C3 (*p* = 0.0009 and *p* = 0.0005, respectively) (Figure 2e). Regarding MRSA *S. aureus* O, the inhibitory activity against biofilm formation shown by the EOs tested alone and in association with each other and combined with oxacillin gave excellent results, notably the EEO/TTO association (*p* = 0.0006) (Figure 2f).

### 2.4. Effect of EOs on Multi-Species Biofilm Formation

**Figure 3:**
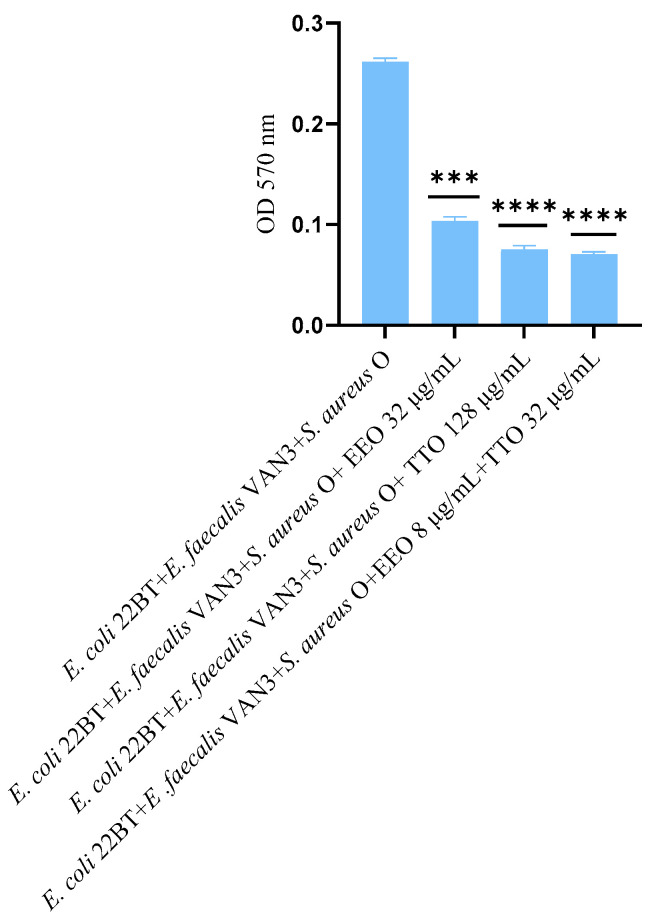
Anti-multi-species-biofilm formation activity of *Melaleuca alternifolia* (TTO), *Eucalyptus globulus* (EEO) EOs and of the association EEO/TTO. Results were expressed in optical density (OD) 570 nm as the arithmetic mean of the three determinations. *p*-values of <0.05 (*), *p* < 0.01 (**), *p* < 0.001 (***) and *p* < 0.0001 (****) were considered significant by *t*-test and ANOVA.

The effect of EOs alone and of the best synergistic mixtures (selected using the highest MIC value obtained for the individual strains) on biofilm in formation by mixed cultures, chosen one per strain (*E. coli* 22BT, *E. faecalis* VAN3, *S. aureus* O), and evaluated with the measurement of the optical density (OD), is shown in Figure 3. Once again, TTO was the best anti-biofilm compound (reduction of 63.08%) (*p* < 0.0001), compared to EEO and the synergistic EEO/TTO association (reduction of 65.38%) (*p* < 0.0001) allowed the obtaining of similar results to the TTO alone but using 1/4 of the MIC value of the single compounds.

### 2.5. Quantification of Viable Cells in Multi-Species Biofilm Formation

**Figure 4:**
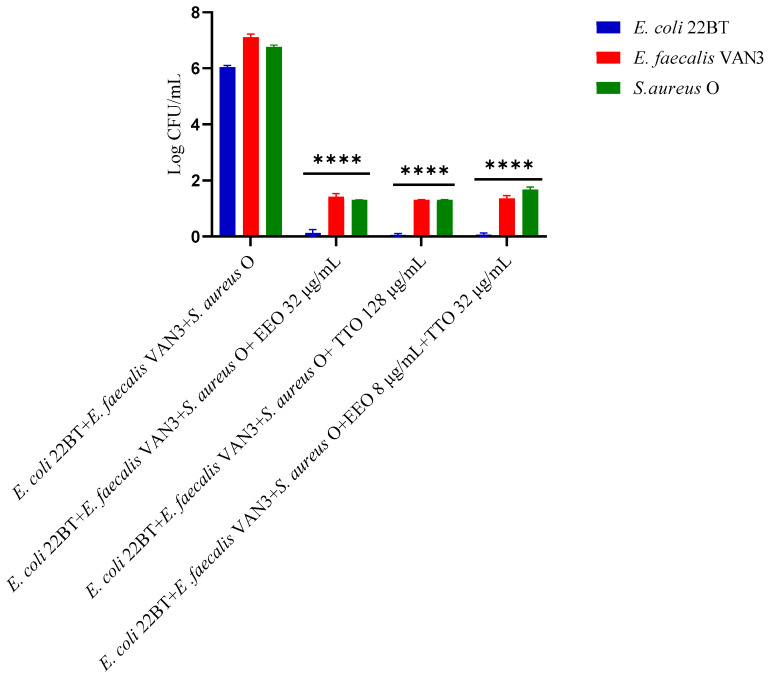
Quantification of viable cells in multi-species biofilm formation using *Melaleuca alternifolia* (TTO), *Eucalyptus globulus* (EEO) EOs and the association EEO/TTO. Results were expressed in Log CFU/mL as the arithmetic mean of the three determinations. *p*-values of <0.05 (*), *p* < 0.01 (**), *p* < 0.001 (***) and *p* < 0.0001 (****) were considered significant by ANOVA test.

To determine the viability of the three ARB within the mixed biofilm in formation and in contact with the EOs alone or in synergistic associations (selected using the highest MIC value obtained for the individual strains), the viable count of the single strains, chosen one per species, was performed on the respective selective media and expressed in CFU (colony forming unit). As shown in Figure 4, the anti-biofilm activity of both the EOs was confirmed even in the case of multi-species biofilm, in particular, against *E. coli* 22BT, and for the latter resulted in a total prevention of its development. The synergistic association TTO/EEO also confirmed the good activity which had emerged in the previous determinations and, even in this case, using a lower concentration than the single EOs.

### 2.6. Effect of EOs on Mono-Species Mature Biofilm

**Figure 5:**
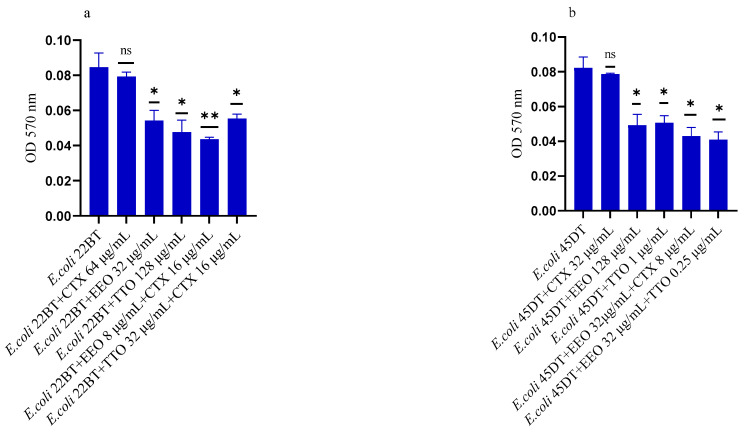

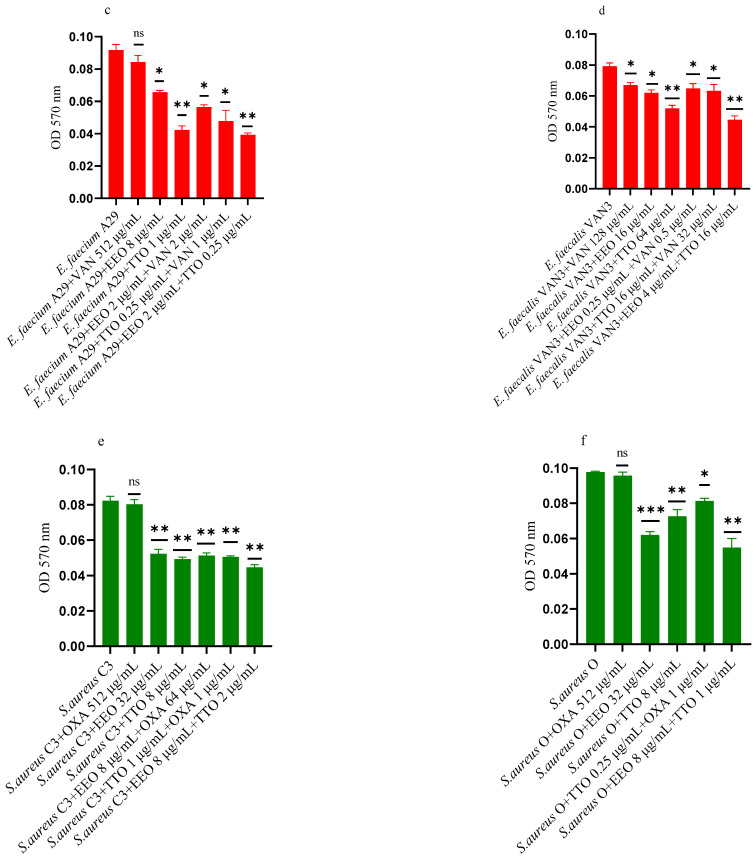
Anti-mature biofilm activity of *Melaleuca alternifolia* (TTO) and *Eucalyptus globulus* (EEO) EOs, of antimicrobials (cefotaxime -CTX-, vancomycin –VAN- and oxacillin-OXA-), and of different combinations/association (antimicrobial/EO and EO/EO) against (**a**) ESBL *Escherichia coli* 22 BT, (**b**) ESBL *Escherichia coli* 45DT, (**c**) VRE *Enterococcus faecium* A29, (**d**) VRE *Enterococcus faecalis* VAN3 (**e**) MRSA *Staphylococcus aureus* C3 and (**f**) MRSA *Staphylococcus* aureus O strains. Results were expressed in optical density (OD) 570 nm as the arithmetic mean of the three determinations. *p*-values of <0.05 (*), *p* < 0.01 (**), *p* < 0.001 (***) were considered significant by *t*-test and ANOVA. using statistical program GraphPad Prism 9.2.0 (San Diego, CA, USA). ns stands for not statistically significant.

Figure 5a–f shows the anti-biofilm activity, referred to mono-species mature biofilms, of the reference antibiotics, of the single EO and of the most advantageous synergistic combinations/associations, which emerged with the FIC index.

Against ESBL *E. coli* 22BT (Figure 5a), TTO was more effective than EEO, and its activity is maintained when combined with cefotaxime (*p* < 0.01) but using lower concentrations than the single compounds. On the strain ESBL *E. coli* 45DT (Figure 5b) 1/4 MICs of the association EEO/TTO and combination EEO/cefotaxime could reduce the mature biofilm.

As regards VRE *E. faecium* A29 (Figure 5c), TTO produced very effective results (*p* = 0.005) against the mature biofilm, and the concentrations needed were 4 times lower when used in association with EEO (*p* = 0.002). It is interesting to note that the combination TTO/VAN determined a renewed sensitivity of the strain to the reference antibiotic, which demonstrated its effectiveness at concentrations reduced 500-fold.

TTO was also more effective than EEO against the mature biofilm of VRE *E. faecalis* VAN3 (Figure 5d), while the combinations EEO/VAN and TTO/VAN exhibit the same activity, even if at reduced compounds concentration. Against this strain, the best anti-mature biofilm activity was shown by the EEO/TTO association (*p* = 0.0056).

Lastly, both EOs proved to be excellent candidates in inhibiting mature biofilm of both MRSA strains (Figure 5e,f), far exceeding the effectiveness of the reference antibiotic. EEO/oxacillin and TTO/oxacillin combinations could reduce the mature biofilm at lower concentration than the single compounds and, even in this case, determined a renewed sensitivity of the strain to the reference antibiotic. Once again, the association EEO/TTO has led to a significant reduction of the mature biofilm at a lower concentration than the single compounds (*p* = 0.0038 and *p* = 0.0046 for *S. aureus* C3 and *S. aureus* 0, respectively).

### 2.7. Effect of EOs on Multi-Species Mature Biofilm

**Figure 6:**
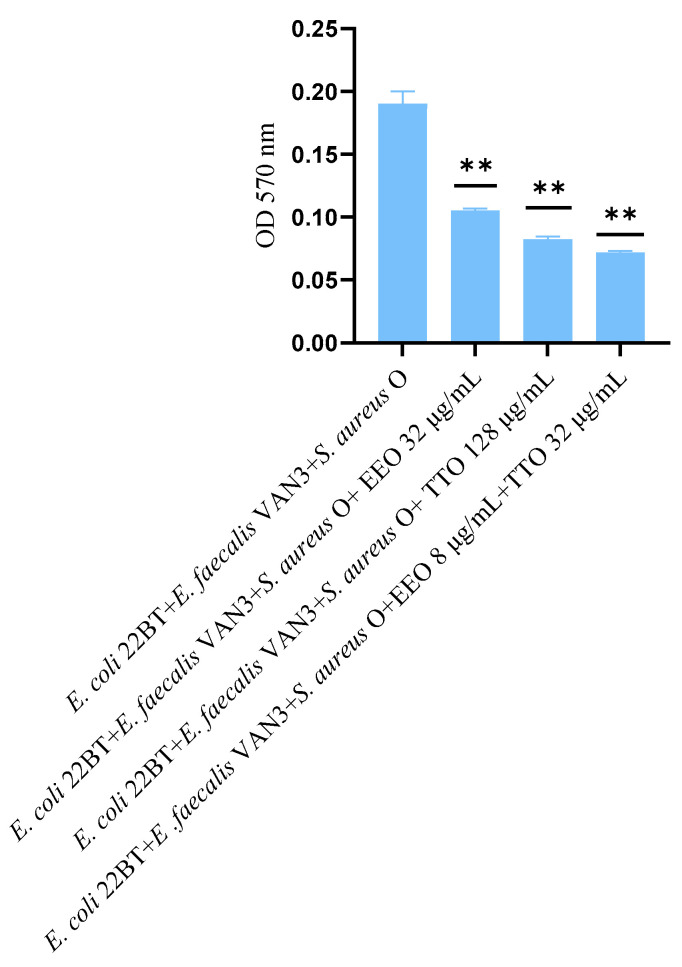
Anti- multi-species mature biofilm activity of *Melaleuca alternifolia* (TTO), *Eucalyptus globulus* (EEO) EOs and the association EEO/TTO. Results were expressed in optical density (OD) 570 nm as the arithmetic mean of the three determinations. *p*-values of <0.05 (*), *p* < 0.01 (**), *p* < 0.001 (***) and *p* < 0.0001 (****) were considered significant by *t*-test and ANOVA.

The effect of EOs alone and of the best synergistic mixtures (selected using the highest MIC value obtained for the individual strains) on multi-species mature biofilms, chosen one per strain (*E. coli* 22BT, *E. faecalis* VAN3, *S. aureus O*), and evaluated with the measurement of the optical density (OD), is shown in Figure 6.

The effectiveness of TTO (*p* = 0.004) was better than EEO (*p* = 0.006) in removing multi-species biofilms. The TTO/EEO association (*p* = 0.003) once again demonstrated a synergistic effect, which allowed the obtaining of results similar to TTO, but using lower concentrations than the single EO.

### 2.8. Quantification of Viable Cells in Multi-Species Mature Biofilm

**Figure 7:**
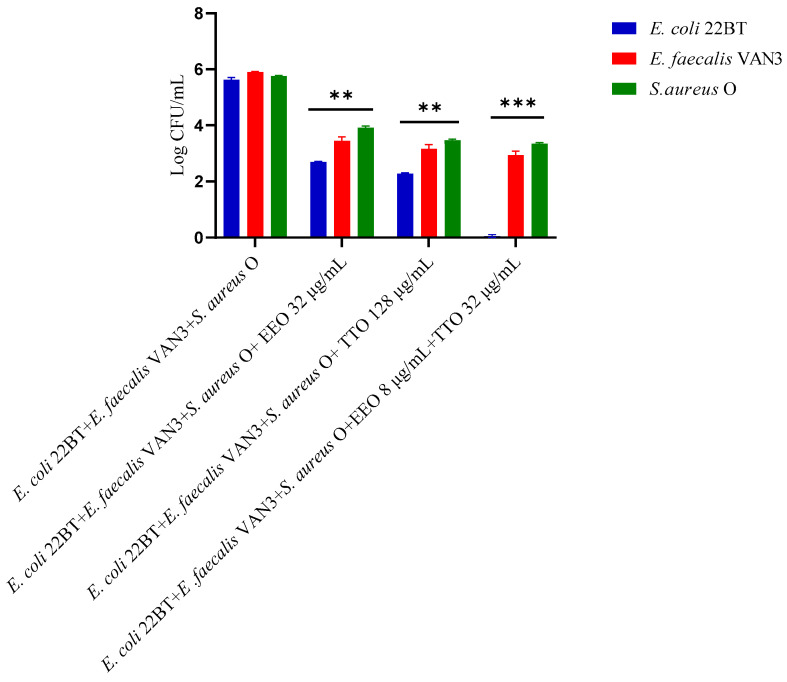
Quantification of viable cells in multi-species mature biofilm using *Melaleuca alternifolia* (TTO), *Eucalyptus globulus* (EEO) EOs and the association EEO/TTO. Results were expressed in Log CFU/mL as the arithmetic mean of the three determinations. *p*-values of <0.05 (*), *p* < 0.01 (**), *p* < 0.001 (***) and *p* < 0.0001 (****) were considered significant by ANOVA test.

To determine the viability of the three ARB within the mixed mature biofilm and in contact with the EOs alone or in synergistic associations (selected using the highest MIC value obtained for the individual strains), the viable count of the single strains, chosen one per species, was performed on the respective selective media and expressed in CFU (Colony Forming Unit). As shown in Figure 7, the anti-biofilm activity of both the EOs was confirmed (*p* < 0.0001). Once again *E. coli* 22BT was found to be the most sensitive strain, as already highlighted in the homologous study performed against the mixed biofilm in formation. The total removal of its mature structure was only obtained using the synergistic association TTO/EEO, even in this case, using lower concentrations than the single EOs.

### 2.9. Epifluorescence Microscopy Observation of Mature Biofilm

**Figure 8:**
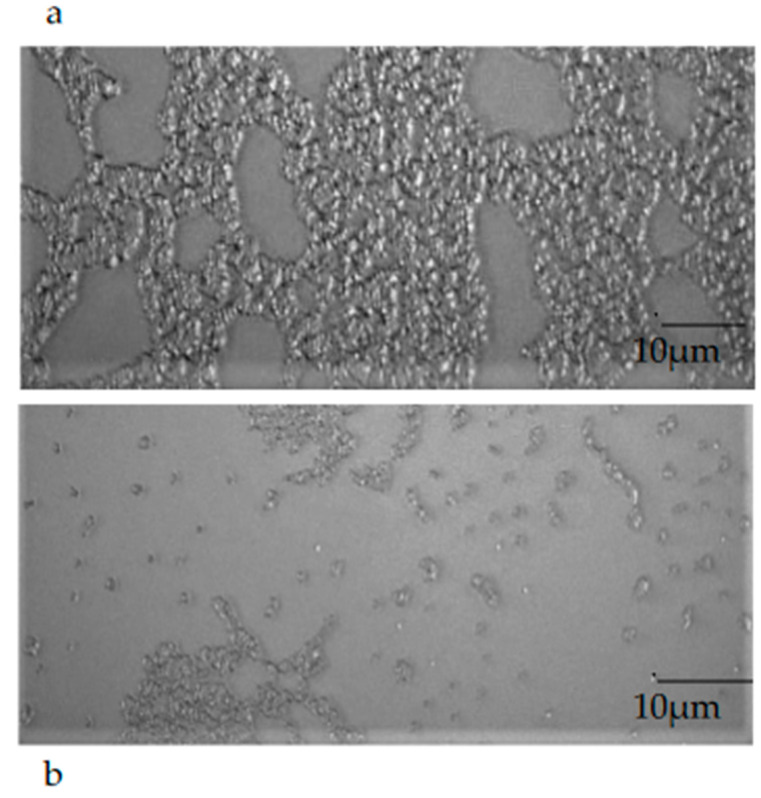
An example of the mature biofilm structure of a *Staphylococcus aureus* O strain in the absence (**a**) and in the presence (**b**) of *Melaleuca alternifolia* essential oil (TTO).

The observation of a mature biofilm produced by the single microorganisms, chosen one per strain (*E. coli* 22BT, *E. faecalis* VAN3, *S. aureus O*), allowed us to evaluate the morphological difference between an intact mature structure and a disrupted and irregular biofilm generated by the strains in contact with EOs (at the respective MIC concentration). This morphological evaluation highlights, once again, the strong anti-biofilm activity of EOs on the mature structure of the biofilm (Figure 8a,b).

## 3. Discussion

In the present investigation, *Melaleuca alternifolia* (Tea-Tree) and *Eucalyptus* EOs, their association or combination with the reference antibiotics were found to be very effective in inhibiting the biofilm produced by ARB bacteria. As regards the biofilm in formation, the best result was obtained against the *E. coli* 22BT biofilm, whose formation was slowed down until it disappeared, as emerged in the quantitative evaluation of multiple bacteria. Less evident are the results obtained on the reduction of mature biofilm produced by the single ARB strains, whereas both EOs have proved to be very effective in reducing the growth of multi-species mature biofilm. The mature biofilm is notoriously a more resistant structure, and these natural antimicrobials, despite their hydrophobic characteristics, which allow their easy diffusion through the polysaccharide membrane, cannot completely penetrate the biofilm meshes and eliminate the consolidated bonds generated during maturation of mono-species biofilm. However, in the presence of a mature biofilm made up of multiple bacteria, other important components within a microbial community could interfere, such as bacterial competition, which may be partly due to the ability of some bacteria to produce biologically active substances (bacteriocins, hemolysins, etc.), capable of destabilizing the structure of the biofilm. In this scenario, the association of several essential oils, in conjunction with the phenomenon of bacterial competition, would seem to allow a deeper penetration into the biofilm and greater effectiveness against this microbial structure. As regards the synergistic effects highlighted during the study of EOs combined with antibiotics, these could be linked to the mechanism of action of essential oils, capable of interacting with the bacterial cell wall, thus facilitating the action of antibiotics or causing cellular autolysis, with coagulation of the cytoplasm and inhibition of glucose-dependent respiration, as reported for TTO [41]. In conclusion, the results obtained in the present investigation could suggest an alternative approach to address the problem of antibiotic resistance using essential oils, better if in association, as adjuvants in antibiotic therapy. Several studies have reported the antibacterial and antibiofilm activity of TTO and EEO, properties believed to be mainly due to the presence of its main components terpinen-4-ol [42,43] and 1,8-cineole [27,44], respectively.

In all tests, the synergistic associations EEO/TTO proved to be more effective than the EOs tested individually, confirming that the biological action of these natural compounds is probably related to the synergy between the different components, and not only to the action of their main constituent, with consequent improvement of the effectiveness of their anti-biofilm activity. The combination of conventional antibiotics and EOs could also represent a possible tool to counter infections caused by ARB strains, notoriously more insidious when incorporated into biofilms. The present study has described and consolidated the synergistic outcome already observed between essential oils and antibiotics. Their combined use has shown the capability to reduce the antibiotic concentrations of employees and, consequently, to decrease the toxic effects during the therapy. These results highlight the ability of essential oils to be a potential modulating agent of antibiotic resistance, as has already emerged from other studies [45,46,47,48,49]. The use of natural antimicrobial agents is increasingly expanding, also thanks to new biotechnologies, such as the incorporation of essential oils in polymeric nanoparticles [50], a technology used to obtain an increase in antibacterial and antioxidant activity, with a reduction of toxic effects and a better penetration into the biofilm [51]. One hope for the future is that this new generation of natural antimicrobials can lead to the development of new drug regimens in the fight against antibiotic resistance.

## 4. Strength and Limits of Research

This study has highlighted the synergies of the EO/EO associations and EO/antibiotics combinations, when used against biofilms produced by clinically important ARB species. The reduction in the concentrations of each component and, in the case of EO/antibiotics combinations, the restoration of the strain’s sensitivity to the reference antibiotics, are interesting results and a starting point for our future investigation. In fact, it is essential to first determine whether the EOs are effective against ARB species, then, eventually, evaluate how many and which active compounds present in the two essential oils are mostly involved in the anti-biofilm activity. It is widely reported in the literature that the concentration of compounds within an essential oil can differ according to the place of production, extraction, part of the plant used, etc. [52]. This may partially represent the limit of the research, whose encouraging results, however, represent a stepping stone for studies more specifically aimed at using the associations of two or more prominent active compounds obtained from both EOs.

## 5. Materials and Methods

### 5.1. Microbial Strains and Essential Oils

All the strains, 2 ESBL *E. coli*, 2 VRE and 2 MRSA (Table 3) were isolated in the Provincial Laboratory of Clinical Microbiology ‘S. Agostino-Estense’ Hospital of Modena (Poirino, Torino, Italy), confirmed by matrix-assisted laser desorption ionization (MALDI) time-of-flight mass spectrometry (TOF/MS) and maintained in the same media containing 20% (*w*/*v*) glycerol at −80 °C until use.

EOs samples from *Melaleuca alternifolia* Chell (Tea-tree) (batch 109 18) and *Eucalyptus globulus* Labill. (batch 067 22), obtained by hydro-distillation (Erboristeria Magentina S.r.l, Poirino, Torino, Italy), were purchased from a local herbalist’s shop in Modena, Italy. *Melaleuca alternifolia* Chell and *Eucalyptus globulus* Labill. plant materials were collected from Australia and Spain and the essential oils (EOs) were extracted from aerial parts of the plant and leaf parts, respectively. These EOs were chosen because their best antibacterial capability emerged from susceptibility testing in a previous investigation carried out on a larger number of MDR strains [40] and summarized (recapped) in Table 3.

### 5.2. GC-MS Analysis

Analyses were performed on a 7890A gas chromatograph coupled with a 5975C network mass spectrometer (Agilent Technologies, Waldbronn, Germany). Compounds were separated on an Agilent Technologies HP-5 MS cross-linked poly-5% diphenyl–95% dimethyl polysiloxane (30 m × 0.25 mm i.d., 0.25 μm film thickness) capillary column. The column temperature was initially set at 45 °C, then increased at a rate of 2 °C/min up to 100 °C, then raised to 250 °C at a rate of 5 °C/min and finally held for 5 min. The injection volume was 0.1 μL, with a split ratio 1:20. Helium was used as the carrier gas, at a flow rate of 0.7 mL/min. The injector, transfer line and ion-source temperature were 250, 280 and 230 °C, respectively. MS detection was performed with electron ionization (EI) at 70 eV, operating in the full-scan acquisition mode in the m/z range 40–400. EOs were diluted 1:20 (*v*/*v*) with n-hexane before GC-MS analysis. All reference standards used for GC analysis, chromatographic grade organic solvents and reagents were purchased from Sigma-Aldrich (Via Monte Rosa, Milan, Italy).

### 5.3. GC-FID Analysis

GC analyses with flame ionization detector (FID) were carried out on a 7820 A from Agilent Technologies. Compounds were separated on an Agilent Technologies HP-5 cross-linked poly-5% diphenyl–95% dimethyl polysiloxane (30 m × 0.32 mm i.d., 0.25 mm film thickness) capillary column. The temperature program was the same as described above. The injection volume was 0.1 μL in split mode 1:20. Helium was used as the carrier gas at a flow rate of 1.0 mL/min. The injector and detector temperature were set at 250 and 300 °C, respectively. EOs and the reference standards were diluted 1:20 (*v*/*v*) with n-hexane before GC-FID analysis. The analyses were performed in triplicate.

### 5.4. Qualitative and Semi-Quantitative Analysis

Compounds were identified by comparing the retention times of the chromatographic peaks with numerous authentic reference standards run under the same conditions and by comparing the IRIs relative to C8–C40 n-alkanes obtained on the HP-5 column under the above-mentioned conditions with the literature [53]. Peak enrichment by co-injection with authentic reference compounds was also carried out. Comparison of the MS-fragmentation pattern of the target analytes with those of pure components was performed. A mass-spectrum database search was carried out by using the National Institute of Standards and Technology (NIST, Gaithersburg, MD, USA) mass-spectral database (version 2.0d, 2005). Semi-quantification was calculated as the relative percentage amount of each analyte, in particular the values were expressed as the percentage peak area relative to the total composition of each EO obtained by GC-FID analysis.

### 5.5. The Minimum Inhibitory Concentration (MIC) of EOs and Antibiotics

The MIC values of both antibiotics and EOs were determined against all microorganisms by the broth microdilution method in 96-well microplates, according to the Clinical Laboratory Standards Institute (CLSI) guidelines 2019 [54], using as antibiotics oxacillin, vancomycin and cefotaxime for MRSA, VRE and *E. coli* ESBL, respectively. The test was performed in sterile 96-well microplates by dispensing into each well 95 µL of Tryptic Soy Broth (TSB) (Oxoid S.p.A, Milan, Italy) and 5 µL of bacterial suspensions, to a final inoculum concentration of 10^6^ CFU/mL. Then, 100 µL of EOs serial dilutions were added to obtain concentrations ranging from 512 to 0.125 μg/mL. Negative control wells consisted of bacteria in TSB without antibiotics and EOs. The plates were incubated at 37 °C for 24 h, mixed on a plate shaker at 300 rpm for 20s, and the MIC was defined as the lowest concentration of antibiotics and EOs that inhibited visible growth of the tested microorganisms after optical density (OD) measurement at 570 nm using a microtiter-plate reader. To confirm the presence or absence of viable cells, we used 2,3,5-triphenyltetrazolium chloride (TTC) sterile solution at concentrations of 0.5% (*w*/*v*), at a volume of 20 μL, to reveal bacterial growth, after incubation at 37 °C for a further 2 h. After the incubation, it was possible to distinguish the live samples that changed from yellow to red. All the experiments were carried out in triplicate, and results were expressed as the arithmetic mean of the three determinations.

### 5.6. Determination of the Fractional Inhibitory (FIC) Index

Using the fractional inhibitory concentration (FIC) index, EO/EO associations and EO/antibiotic combinations were used to analyze their activity toward the six MDR strains, chosen, two per genera, because of their sensitivity, which emerged in a previous study. The antimicrobial assays were performed using the checkerboard method with a 96-well microplate [35], and the FIC index was calculated by comparing the value of the MIC of each agent alone with the combination-derived MIC. The results were considered as synergy (FIC ≤ 0.5), addition (0.5 ≤ FIC ≥ 1), indifference (1 ≤ FIC ≥ 4), and antagonism (FIC > 4). The experiments were conducted in the same manner as for the MIC determination in the susceptibility testing.

### 5.7. EOs Activity on Biofilm Produced by Single and Mixed Strains

The capability of all the ARB strains to form biofilm was studied using a modified 96-well microtiter-plates method [55]. The effects of EOs, antibiotics, and of the EO/EO associations and EO/antibiotic combinations in 24 h formed biofilm were evaluated according to an adapted Kwieciński et al. (2009) method [51]. For each microorganism, an overnight culture (18/24 h) was diluted in fresh sterile TSB to the final concentration of 10^6^ CFU/mL, and 150 μL single cultures or 50 μL for each strain in the mixture were dispensed into each well of a 96-well plate, and incubated for 24h at 37 °C. (i) to determine the activity against mature biofilm, planktonic cells were aspirated carefully, and the wells were washed three times with sterile phosphate-buffered saline (PBS, pH 7.2). The compounds were then added at the respective MIC of each strain, and for both association EO/EO and combination EO/antibiotic at the best synergistic concentrations, as previously detected by the measurement of the FIC index. The plates were then incubated for 24 h at 37 °C. (ii) For the study of the effectiveness on biofilm formation, the compounds were added at the same time together with the suspensions and incubated for 24 h at 37 °C. Sterile TSB was used as negative control. In both cases, the biofilm biomass was quantified according to the crystal violet staining method by Stepanovic et al. (2000) [56]. After incubation, plates were washed three times with a sterile phosphate-buffered saline solution (PBS, pH 7.2) to remove planktonic bacteria, and fixed with 200 μL of 99% methanol for 15 min. Plates were then emptied, air-dried, and stained with 150 μL of 0.2% of crystal violet solution for 15 min at room temperature. After staining, the wells were washed three times with sterile PBS, and the dye bound to the cells was dissolved in 33% glacial acetic acid (Sigma-Aldrich, Saint Louis, MO, USA). Results were expressed in terms of optical density (O.D.) values at 570 nm, using a microplate reader (Sunrise Tecan, Grödig, Austria), as the arithmetic mean of the three determinations, and the standard deviation was reported as error bars.

### 5.8. Quantification of Viable Cells in Multi-Species Biofilm

The quantification of viable cells in multi-species biofilms, grown as above in the microtiter-plates, was determined using the plate counting technique. For removal of cells from the bottom of the wells, plates were carefully washed three times with a sterile PBS and 1 mL of PBS was added to each well. Using a sterile pipette tip, the biofilm was scraped. The number of viable cells was determined using MacConkey agar, Brilliance MRSA agar, and Brilliance VRE agar (Oxoid LTD, Basingstoke, UK) for *E. coli*, *S. aureus*, and *Enterococcus* spp., respectively. The Petri dishes were incubated at 37 °C for 24 h. The values were expressed as CFU cm^2^. Assays were performed in triplicates.

### 5.9. Epifluorescence Microscopy Observation of Mature Biofilm

Epifluorescence microscopy was used to evaluate the effectiveness of EOs in controlling previously obtained ARB mature biofilm, following the same methodology described above. Sterile PVC coverslips were placed inside the microtiter-plate wells and, after incubation for 24 h at 37 °C, the wells were washed three times with a sterile phosphate-buffered saline solution (PBS, pH 7.2) to remove planktonic bacteria and the compounds were added at MIC and/or at the chosen synergic concentration. The plate was incubated again at 37 °C for 24 h. Finally, washing with buffer solution (PBS) was performed for each well to remove suspended cells. The coverslips were gently extracted from the plate, dried, fixed with 4% paraformaldehyde and placed on glass slides. Coverslips of untreated biofilm were used as control. The biofilm observation was performed at 40× magnification, under an epifluorescence microscope Nikon eclipse 90 I (Mississauga, Ontario Canada).

### 5.10. Statistical Analysis

Each experiment was replicated three times. The statistical significance was determined by *t*-test and ANOVA test using statistical program GraphPad Prism 9.2.0 (San Diego, CA, USA). *p*-values were considered significant at ≤ 0.05.

## Figures and Tables

**Table 1:** Qualitative and semi-quantitative analysis of *Malaleuca alternifolia* Chell (TTO) and *Eucalyptus globulus* Labill. (EEO) EOs. Data are expressed as % relative peak area values ± standard deviation (SD).

	^a^ Lit. RI	^b^ Exp. RI	*Melaleuca alternifolia*	*Eucalyptus globulus*
α-thujene	928	926	0.88 ^c^	-
α-pinene	936	932	2.56 ± 0.1	2.33 ± 0.1
camphene	950	947	-	0.51 ^c^
sabinene	973	972	0.07 ^c^	-
β-pinene	978	975	0.73 ^c^	0.99 ^c^
β-myrcene	989	991	0.80 ^c^	0.83 ^c^
α-phellandrene	1004	1004	0.45 ^c^	0.23 ^c^
α-terpinene	1017	1017	8.89 ± 0.2	0.09 ^c^
*p*-cymene	1024	1024	2.84 ± 0.1	-
limonene	1029	1028	1.88 ± 0.1	0.93 ^c^
1,8-cineole	1032	1030	2.35 ± 0.1	58.07 ± 0.2
*cis*-β-ocimene	1038	1039	-	0.21 ^c^
γ-terpinene	1060	1059	20.16 ± 0.2	1.11 ^c^
linalool	1099	1100	0.49 ^c^	12.05 ± 0.2
camphor	1143	1146	-	4.39 ± 0.1
borneol	1166	1167	-	1.00 ^c^
terpinen-4-ol	1177	1179	43.29 ± 0.2	0.66 ^c^
α-terpineol	1190	1192	2.99 ± 0.1	0.71 ^c^
linalyl acetate	1263	1264	-	10.95 ± 0.2
α-cubebene	1351	1353	0.06 ^c^	-
α-copaene	1376	1379	0.10 ^c^	-
α-gurjunene	1409	1414	0.32 ^c^	-
β-caryophyllene	1420	1426	0.32 ^c^	0.98 ^c^
aromadendrene	1440	1445	1.07 ^c^	-
α-humulene	1453	1460	0.09 ^c^	0.22 ^c^
allo-aromadendrene	1460	1467	0.47 ^c^	-
germacrene D	1481	1488	-	0.11 ^c^
α-selinene	1493	1496	0.14 ^c^	-
ledene	1495	1501	1.31 ^c^	-
δ-cadinene	1523	1530	1.03 ^c^	-
globulol	1582	1586	0.06 ^c^	-
caryophyllene oxide	1589	1593	0.19 ^c^	0.06 ^c^
viridiflorol	1591	1601	0.17 ^c^	-
Total identified			97.07	96.55

^a^ Literature retention indices (HP-5 MS column) according to NIST 14 (National Institute of Standards and Technology, USA; 14th edition) library database [28], Babushok et al. [29], and Dong et al. [30] (https://webbook.nist.gov, accessed on 27 January 2023); ^b^ Experimental retention indices (HP-5 ms column); ^c^ SD < 0.05.

**Table 2:** MIC (μg/mL) of antimicrobials alone, and of EO/EO association, and EO/antibiotic combination against ARB pathogens in planktonic form. DLC: Drug Lowering Concentration. EEO: eucalyptus, TTO: tea-tree, CTX: cefotaxime, VAN: vancomycin, OXA: oxacillin and n.s.: no synergy.

Strains	Drug/EO	MIC Alone (μg/mL)	MIC EO/EO and EO/Drug (μg/mL)	DCL	Strains	Drug/EO	MIC Alone (μg/mL)	MIC EO/EO and EO/Drug (μg/mL)	DCL
*E. coli* 22BT					*E. coli* 45DT				
	CTX EEO	64 32	16 8	4-fold 4-fold		CTX EEO	32 128	8 32	4-fold 4-fold
	CTX TTO	64 128	16 32	4-fold 4-fold		CTX TTO	n.s	n.s	n.s
	EEO TTO	n.s	n.s	n.s		EEO TTO	128 1	32 0.25	4-fold 4-fold
*E. faecium* A29					*E. faecalis* VAN3				
	VAN EEO	512 8	2 2	256-fold 4-fold		VAN EEO	128 16	0.5 0.25	256-fold 64-fold
	VAN TTO	512 1	1 0.25	512-fold 4-fold		VAN TTO	128 64	32 16	4-fold 4-fold
	EEO TTO	8 1	2 0.25	4-fold 4-fold		EEO TTO	16 64	4 16	4-fold 4-fold
*S. aureus* C3					*S. aureus* O				
	OXA EEO	512 32	64 8	8-fold 4-fold		OXA EEO	n.s	n.s	n.s
	OXA TTO	512 8	1 1	512-fold 8-fold		OXA TTO	512 8	1 0.25	512-fold 32-fold
	EEO TTO	32 8	8 2	4-fold 4-fold		EEO TTO	32 8	8 1	4-fold 8-fold

**Table 3:** Strains and respective MIC of EOs and antibiotics used in the study. EEO: eucalyptus, TTO: tea-tree, CTX: cefotaxime, VAN: vancomycin, OXA: oxacillin and n.a.: not adaptable.

Strains	EEO (µg/mL)	TTO (µg/mL)	CTX (µg/mL)	VAN (µg/mL)	OXA (µg/mL)
*Escherichia coli* 22BT	32	128	64	n.a	n.a
*Escherichia coli* 45DT	128	1	32	n.a	n.a
*Enterococcus faecium* A29	8	1	n.a	512	n.a
*Enterococcus faecalis* VAN 3	16	64	n.a	128	n.a
*Staphylococcus aureus* C3	32	8	n.a	n.a	512
*Staphylococcus aureus* O	32	8	n.a	n.a	512

## Data Availability

Not applicable.

## Supplementary Materials

The following supporting information can be downloaded at: https://www.mdpi.com/article/10.3390/molecules28041671/s1, Figure S1: GC chromatograms of pure (a) and spiked (b) *Melaleuca alternifolia* essential oil with most abundant mono- and sesquiterpenes (c). Figure S2: GC chromatograms of pure (a) and spiked (b) *Eucaliptus globulus* essential oil with most abundant mono- and sesquiterpenes (c).
