# Supplementary material for: Effects of Melaleuca alternifolia Chell (Tea Tree) and Eucalyptus globulus Labill. Essential Oils on Antibiotic-Resistant Bacterial Biofilms
Source: Molecules. 2023 Feb 9;28(4):1671. doi: 10.3390/molecules28041671 (PMC9961662; doi:10.3390/molecules28041671)
Supplement: Supplementary file 1 [file molecules-28-01671-s001.zip › molecules-2161168-supplementary.pdf]

Article

Effects of *Melaleuca alternifolia* Chell (Tea Tree) and *Eucalyptus globulus* Labill. Essential Oils on Antibiotic-Resistant Bacterial Biofilms

Ramona Iseppi [1], Martina Mariani [2], Stefania Benvenuti[1], Eleonora Truzzi [1] and Patrizia Messi [1]*

[1] Department of Life Sciences, University of Modena and Reggio Emilia, Via G. Campi 287, 41125 Modena, Italy

[2] Department of Emergency Surgery, Anesthesia and Intensive Care Unit, A.O.R.N. Antonio Cardarelli, via A. Cardarelli 9, 80131 Naples, Italy

* Correspondence: patrizia.messi@unimore.it

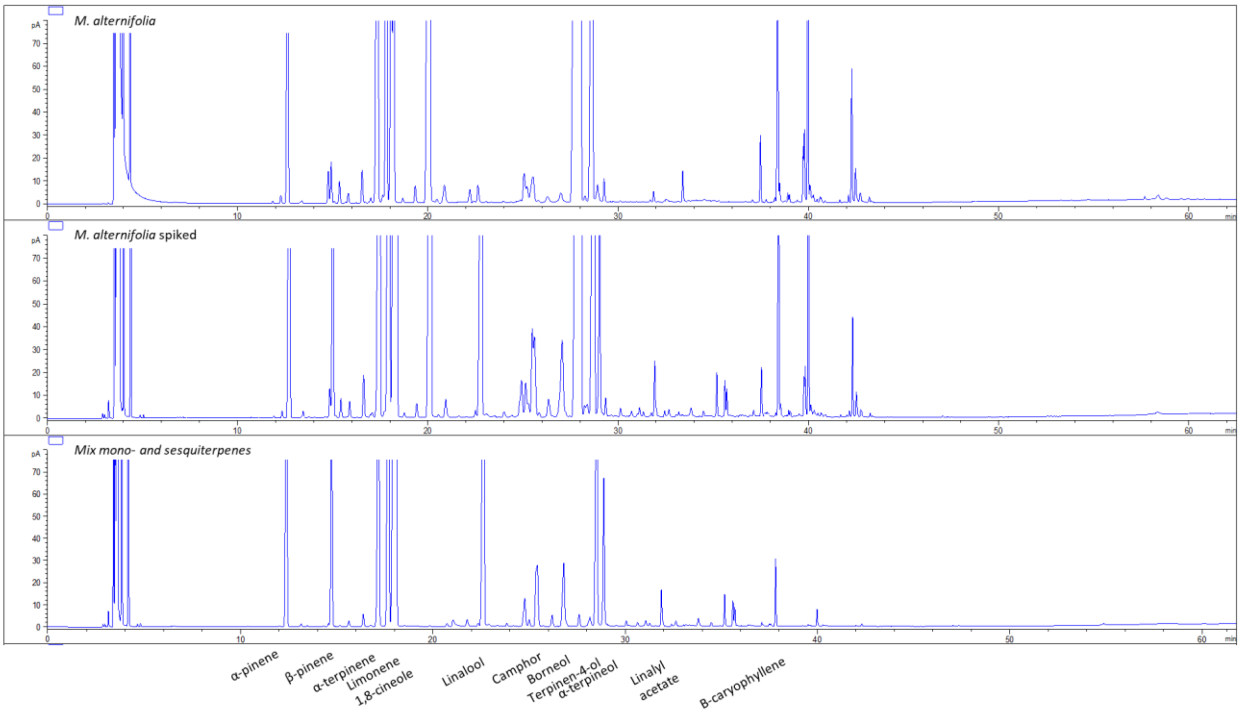

Figure S1: GC chromatograms of pure (a) and spiked (b) *Melaleuca alternifolia* essential oil with most abundant mono- and sesquiterpenes (c).

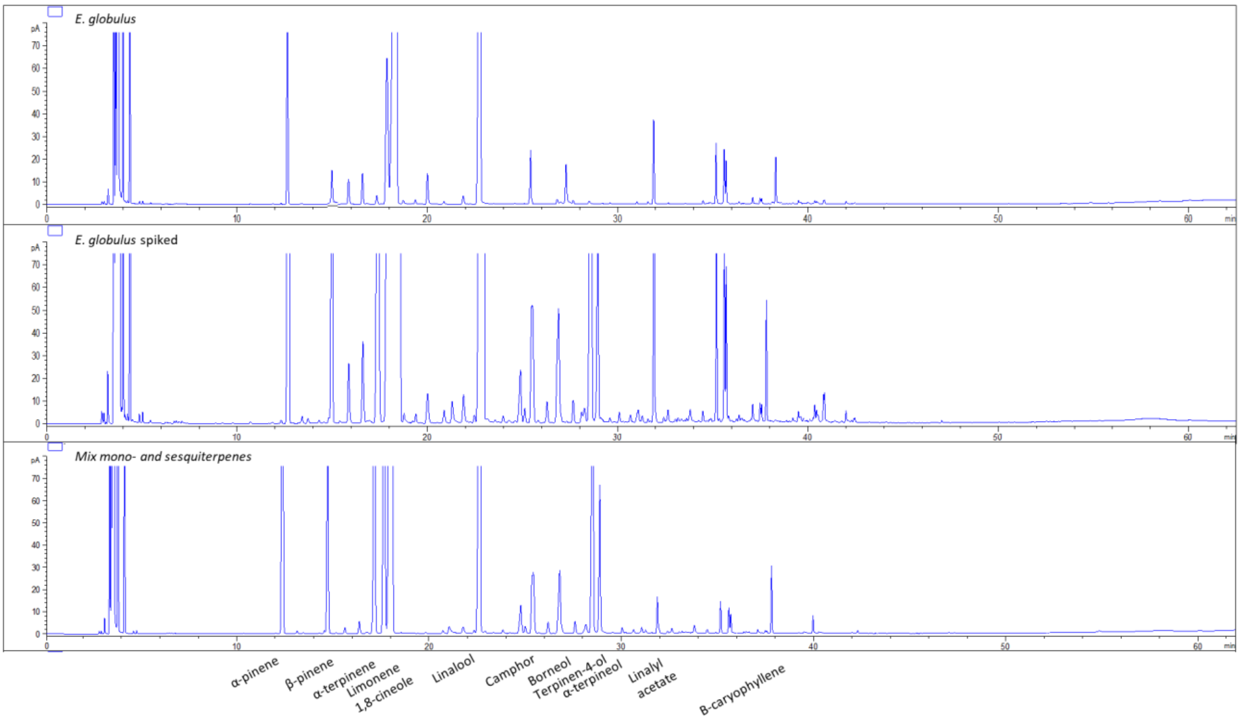

Figure S2: GC chromatograms of pure (a) and spiked (b) *Eucaliptus globulus* essential oil with most abundant mono- and sesquiterpenes (c).
